# Progress of research on graphene and its derivatives in bone and cartilage repair

**DOI:** 10.3389/fbioe.2023.1185520

**Published:** 2023-06-08

**Authors:** Shilong Yu, Mingke You, Kai Zhou, Jian Li

**Affiliations:** ^1^ West China School of Medicine, West China Hospital, Sichuan University, Chengdu, China; ^2^ Sports Medicine Center, West China Hospital, Sichuan University, Chengdu, China; ^3^ Department of Orthopedics, Orthopedic Research Institute, West China Hospital, Sichuan University, Chengdu, China

**Keywords:** graphene-based materials, bone, cartilage, tissue engineering, nanobiocatalysis

## Abstract

In recent years, graphene and its derivatives have gained wide attention in the biomedical field due to their good physicochemical properties, biocompatibility, and bioactivity. Its good antibacterial, osteoinductive and drug-carrying properties make it a promising application in the field of orthopedic biomaterials. This paper introduces the research progress of graphene and its derivatives in bone tissue engineering and cartilage tissue engineering and presents an outlook on the future development of graphene-based materials in orthopedics.

## 1 Introduction

Artificial joint replacement is mainly used for serious deformities of joint structures caused by arthritis, fractures, benign and malignant tumors, etc., which can relieve pain, correct deformities, and restore or improve function. Among all replacement procedures, the demand for knee and hip replacements is the strongest. In mainland China, there were 2,531,341 cases of total hip arthroplasty (THA) and 1,369,950 cases of total knee arthroplasty (TKA) between 2011 and 2019 (Feng B. et al., 2021). In the US, 2,244,587 primary and revision hip and knee arthroplasties were performed from 2012 to 2020 (Siddiqi et al., 2022). The strong demand for artificial joint replacement has also brought attention to the innovation of artificial joint materials, in which bone and cartilage tissue engineering is closely related to providing safer, more stable and functional artificial joints *in vivo*. As an emerging excellent nanobiomaterial, graphene-based materials have received great attention in major fields such as chemistry, physics, materials, and medical biology (Geim and Novoselov, 2007). Boccaccini *et al.* discovered that carbon nanotubes (CNTs) have great applications in biomedicine (Boccaccini and Gerhardt, 2010). Graphene-based materials in cardiac, neural, skeletal muscle, and skin/adipose tissue engineering have drawn particular attention in recent years (Shin et al., 2016). In regard to bone and cartilage tissue engineering specifically, hybrid scaffolds based on graphene nanomaterials have shown great potential in osteogenesis and chondrogenesis (Kang et al., 2022). These all attract more study on graphene and its derivatives in bone and cartilage tissue engineering.

Graphene is a two-dimensional hexagonal planar polycyclic aromatic hydrocarbon atomic crystal with sp2 hybridized carbon atoms arranged in a honeycomb shape, whose unique atomic structure gives it special chemical, physical, mechanical, thermal, electronic and optical properties. In 2004, Geim and Novoselov isolated graphene from graphite by micromechanical forces, for which they received the Nobel Prize in Physics in 2010 (Novoselov et al., 2004).

Graphene-based materials broadly refer to 2D carbon materials related to graphene, including graphene and its derivatives, such as graphene oxide (GO) and reduced graphene oxide (rGO). Meanwhile, other carbon materials, such as metal carbides and/or nitrides (MXenes) and fullerene (C60) also play an important role. Because of their physicochemical properties of high biocompatibility, low toxicity, high specific surface area, and strong adsorption ability to small molecules, graphene materials are an ideal material for tissue engineering (Nejabat et al., 2017). However, single-layer defect-free graphene is difficult to prepare, while derivatives of graphene, while maintaining the properties of graphene itself, solve the problem of its difficult dispersion in aqueous solutions and organic solvents by introducing functional group modifications (e.g., hydroxyl, carboxyl, epoxy groups, etc., and can be used more widely in biomedical fields by forming biocomposites with other materials. This paper focuses on the latest research progress of graphene materials in the field of bone and cartilage tissue engineering (Table 1).

**TABLE 1:** Summary of the application of graphene and its derivatives in bone and cartilage tissue engineering and nanobiocatalysis.

Materials	Graphene and its derivatives attatched	Characteristics	Function	Application	References
chitosan	GO	improved elastic modulus, tensile strength, and elongation	promoting cellular proliferation	artificial bone materials	Liu et al. (2008), Gao et al. (2014)
polydimethylsiloxane (PDMS)	RGO	good mechanical strength and voids ranging from 10 to 600 μm in diameter	stimulating the differentiation of human ADSCs to the osteoblast lineage		Dinescu et al. (2014)
hydroxyapatite (HA)	rGO	better mechanical strength, hardness and Young’s modulus	enhancing the osteogenic differentiation of cells and new bone formation		Dinescu et al. (2014), Li et al. (2017)
	GO (modified by sulfate groups)		promoting calcium ion aggregation and driving HA mineralizatio	bone repair	Fan et al. (2014)
hierarchical porous HA	rGO		accelerating bone growth and the repair of critical bone defects		Zhou et al. (2019)
poly-methyl methacrylate (PMMA)	GO	enhanced mechanical properties, promoted cell proliferation	binding the bone cement to the adjacent bone		Gonçalves et al. (2013)
ultrahigh molecular weight polyethylene (UHMWPE)	G	increased fracture toughness and tensile strength	reducing aseptic loosening after arthroplasty	arthroplasty	Lahiri et al. (2012)
protenis	G	high nanoscale porosity, excellent protein-bearing capacity	improving cell survival and chondrocyte differentiation	cartilage tissue engineering	Yoon et al. (2014)
chondroitin sulfate/ethylene glycol	GO	bionic three-dimensional environment	improving survival rate of chondrocyte growth		Liao et al. (2015)
N-doped rGO (N-rGO)		good biocompatibility and great stability	enhanceing peroxidase-mimicking activities	tumor catalytic therapy	Hu et al. (2018), Liang et al. (2020)
G encapsulated with TiO2		scavenging free radicals, protecting cells against oxidative stress		anti-oxidation treatment	Qiu et al. (2014)
reactive oxygen species (ROS)-based nanomaterials					Zhou et al. (2020), Zhao et al. (2022)
graphene quantum dots (GQDs)		anti-inflammation abilities		intestinal bowel diseases (IBDs) treatment	Lee et al. (2020)
Metal carbides and/or nitrides (MXenes)		high metallic conductivity, excellent hydrophilicity and a large surface area	oxidase, peroxidase, superoxide dismutase, and catalase activities	nanobiocatalysis	Feng W. et al. (2021), Liu et al. (2022)
tris-malonic acid derivative of fullerene (C60)		superoxide dismutase activities		cardiac damage treatment	Ali et al. (2004), Hao et al. (2017)
GO in gold nanoreaction between HAuCl4 and H2O2		high reaction rate	surface-enhanced Raman scattering (SERS) activity	detection of HCG	Liang et al. (2017)

### 1.1 Special biological properties of graphene and its derivatives

#### 1.1.1 Antibacterial ability

Postoperative periprosthetic infection is a catastrophic complication of bone implant materials. Therefore, the design of bone implant materials with good antibacterial properties and osteogenic function is extremely important in the field of bone tissue engineering. However, the traditional method of loading antibiotics into bone implant materials is increasingly limited by the problems of bacterial biofilms on the surface of the prosthesis blocking the action of drugs and the development of bacterial resistance. Here, the discovery of graphene materials has brought a chance to solve the problem.

Hu *et al.* found that GO has a significant antibacterial effect. It could inactivate *Escherichia coli* within 2 h (Hu et al., 2010). Subsequent studies have reported that GO also shows excellent antibacterial activity against other common Gram-negative or Gram-positive bacteria (Liu et al., 2011; Krishnamoorthy et al., 2012; Tu et al., 2013; Yin et al., 2013). The specific antibacterial mechanism of GO is still inconclusive, but it is generally attributed to two main mechanisms: damage to the cell structure integrity of the exposed bacteria and oxidative stress. The former is mainly a physical damage mechanism: when bacteria come into contact with the GO surface, GO can kill bacteria by damaging the cell membrane through the sharp structure of the edge, extracting phospholipid molecules from the bacterial cell membrane and destroying its structural integrity (Hu et al., 2010; Tu et al., 2013). The latter is mainly a chemical mechanism: after contact with GO, bacterial intracellular reactive oxygen species (ROS) and reduced glutathione (GSH) ratios are imbalanced, resulting in lipid peroxidation, mitochondrial dysfunction, and protein inactivation, which lead to cell apoptosis (Gurunathan et al., 2013).

#### 1.1.2 Bone tissue regeneration promotability

In the process of repair and reconstruction of defective bone tissue, biomaterials not only need to provide the mechanical and physiological environment required for cell adhesion, growth, proliferation, and metabolism but also play an essential role in regulating the differentiation of stem cells to osteoblasts. The good physicochemical properties, biocompatibility and pro-stem cell osteogenic differentiation properties of graphene make it show promising applications in osteoconduction (Kim et al., 2013).

Graphene material coatings are biocompatible and provide good conditions for the adhesion and proliferation of osteogenic-associated fibroblasts, osteoblasts, and mesenchymal stem cells (MSCs) (Ryoo et al., 2010). The stiffness and strain of the stem cell culture substrate are important factors affecting stem cell differentiation (Jang et al., 2011), and the extremely high Young’s modulus and good flexibility to cope with out-of-plane deformation of graphene contribute to its role in promoting osteogenic differentiation of stem cells. McBeath *et al.* have grown human osteoblasts and MSCs on GO and silica surfaces, respectively. After 48 h of culture, they found that the number of proliferations on the graphene surface was significantly higher than the latter, and the morphology of MSCs on graphene was spindle-shaped, different from the irregular polygonal cells on the surface of SiO2 plates (Jang et al., 2011). Considering that MSCs with spindle morphology tend to have a higher potential to differentiate into osteoblasts, this suggests a tendency of osteogenic differentiation of MSCs in the graphene medium (McBeath et al., 2004). Nayak *et al.* found that BMSCs in graphene medium showed significant expression of the osteoblast marker osteocalcin (OCN) and more calcium deposition after alizarin red staining. The effect was similar to that of the classical pro-osteo-differentiation factor bone morphogenetic protein 2 (BMP2) (Kalbacova et al., 2010).

#### 1.1.3 Drug delivery capacity

Graphene has a large specific surface area and can assume a larger drug loading capacity. In addition, the noncovalent π-π bonds and hydrogen bonds in the structure allow graphene to adsorb more proteins and drugs.

In 2008, Liu *et al.* were the first to realize the drug-carrying function of graphene. They loaded an anticancer drug camptothecin derivative (SN38) into PEG-modified GO and demonstrated the good biosafety of graphene materials as drug carriers (Nayak et al., 2011). Yang *et al.* successfully loaded GO with a large amount of adriamycin and demonstrated that the drug-carrying effect of GO was mainly accomplished by π-π stacking (Liu et al., 2008). Zhang *et al.* were the first to report that loading GO with multiple anticancer drugs for mixed transport reduced tumor drug resistance (Yang et al., 2010).

La *et al.* loaded BMP-2 onto GO-coated titanium (Ti) substrates and found that they released a significant amount of BMP-2. The GO-coated Ti substrates were found to promote osteoblast differentiation more than the pure Ti substrates. The study also found that there was more new bone formation in the Ti/GO-BMP2 group after implantation in a mouse cranial defect model. The above study showed that graphene has a large drug loading and controlled release capacity. It can also be used as an excellent drug carrier and slow release material in bone tissue engineering (Yang et al., 2010).

### 1.2 Progress in the application of graphene and its derivatives in bone tissue engineering

The purpose of tissue engineering is to restore or improve the morphology and function of damaged tissues and organs to achieve ultimate reconstruction. Among the three major elements of bone tissue engineering, scaffold material, growth factors and seed cells, scaffold material is an extremely important part. The three-dimensional structure of the material scaffold can provide an ideal microenvironment for cell adhesion and proliferation, mechanically supporting bone regeneration. The main problems of conventional bone tissue engineering scaffold materials currently include insufficient strength and low osteogenic induction capacity. Graphene (G) is a new carbon nanomaterial with great application potential that was discovered recently. The good mechanical strength and electrical conductivity, promotion of cell proliferation and differentiation, and many other biological functions make it a hot research topic in biomedical fields such as tumor therapy and neuromuscular regeneration (Kalbacova et al., 2010).

**FIGURE 1:**
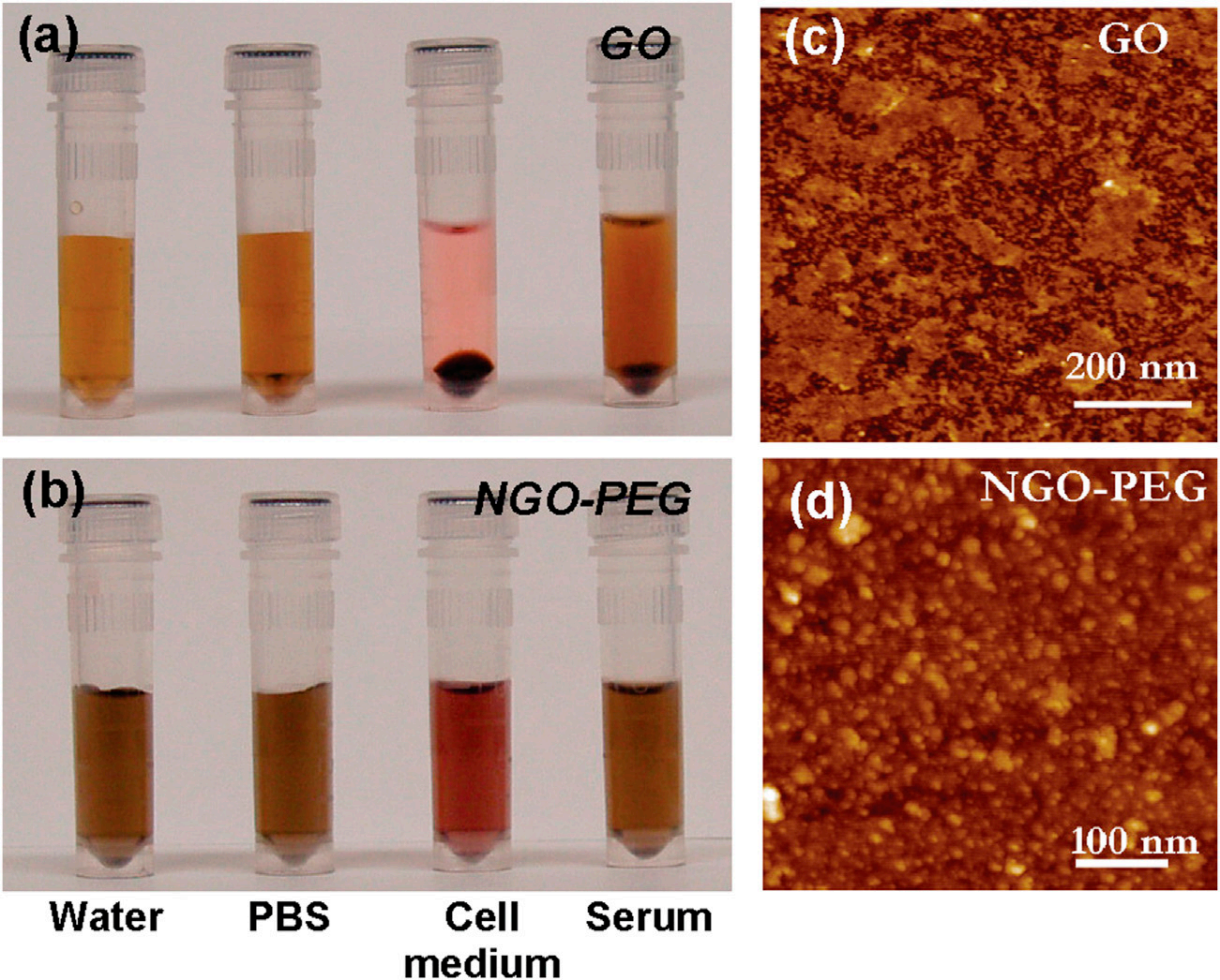
PEGylation of graphene oxide: photos of GO **(A)** and NGO−PEG **(B)** in different solutions recorded after centrifugation at 10000 *g* for 5 min. GO crashed out slightly in PBS and completely in cell medium and serum (top panel). NGO−PEG was stable in all solutions; AFM images of GO **(C)** and NGO-PEG **(D)** (Liu et al., 2008).

Graphene-based materials compounded with conventional bone tissue engineering scaffolds can increase toughness through crack bridging, crack deflection, and crack tip shielding, improving the mechanical properties of conventional materials, thus better helping the original materials achieve bone tissue engineering functions (Zhang et al., 2010). Chitosan is a natural polymer material with good biocompatibility and bioactivity that is rich in functional groups and is highly regarded among biomaterials. However, its poor mechanical properties and low strength limit its application. Recently, a study prepared composites by adding a 1% GO sheet layer to chitosan, which improved the elastic modulus, tensile strength, and elongation by 51%, 93%, and 41%, respectively, compared to pure chitosan (Figure 1 inserted) (Liu et al., 2008). Dinescu *et al.* added chitosan at 0.5% and 3% mass ratios of GO and discovered improvements in scaffold void formation, mechanical properties, and bioactivity. *In vitro* experiments revealed that the material containing 3% GO was more cytocompatible and better able to promote cellular proliferation, which has potential as a 3D scaffold material for bone tissue engineering (Gao et al., 2014).

**FIGURE 2:**
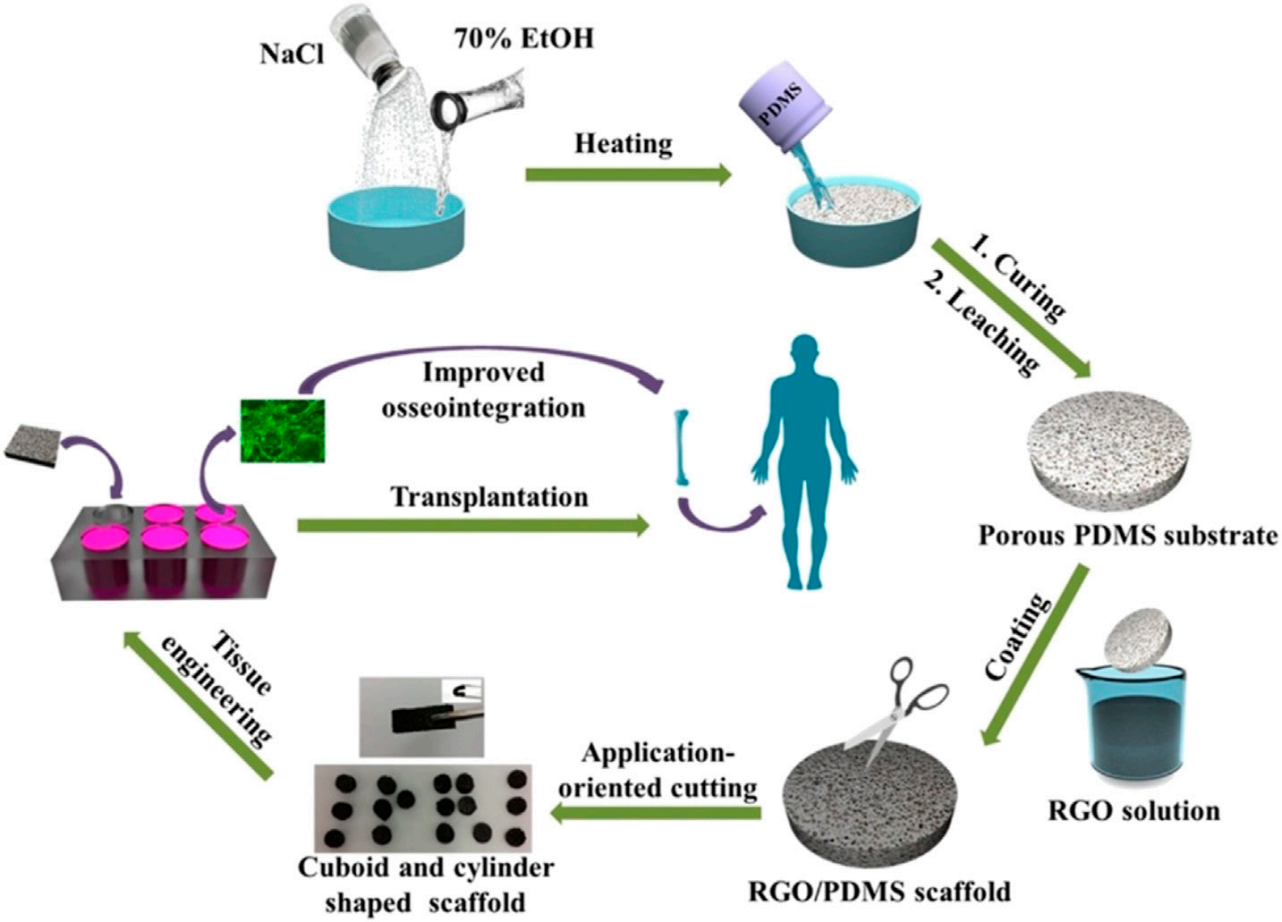
Schematic illustration of the fabrication process and application for improved osseointegration of porous rGO/PDMS scaffold (Li et al., 2017).

Polydimethylsiloxane (PDMS) is a widely used silicon-based organic polymer material whose biocompatibility and high solubility of oxygen make it an ideal material for cell transplantation. However, its high hydrophobicity inhibits PDMS bioactivity, limiting its use in tissue engineering. Recently, a study coated PDMS 3D scaffolds with RGO, which formed RGO/PDMS 3D scaffolds with good mechanical strength and voids ranging from 10 to 600 μm in diameter. The composite 3D scaffold was found to stimulate the differentiation of human adipose stem cells (ADSCs) to the osteoblast lineage, suggesting that the RGO/PDMS 3D scaffold may be a potential osteointegration graft (Figure 2 inserted) (Dinescu et al., 2014).

**FIGURE 3:**
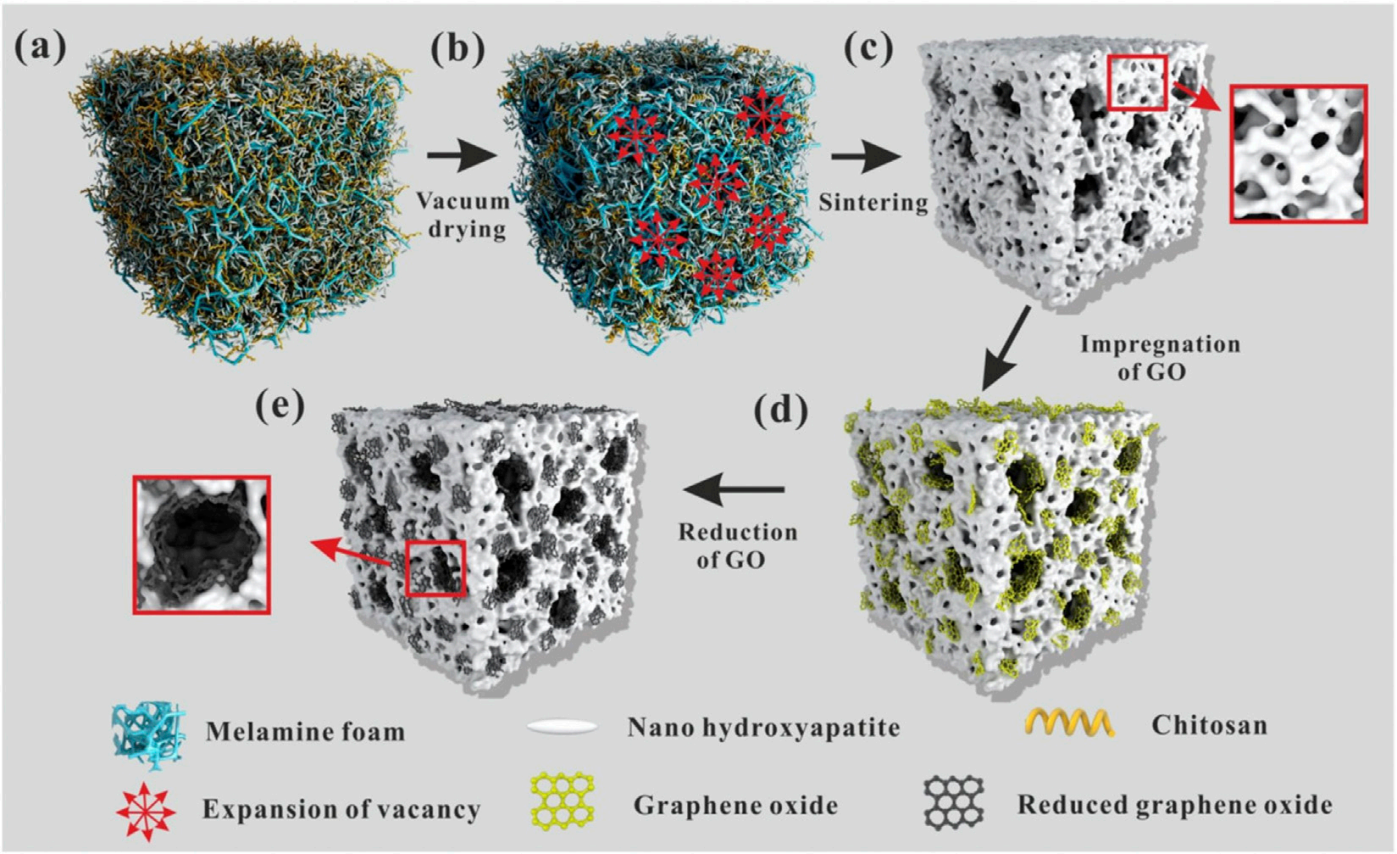
The formation mechanism of the hierarchical pore structure is schematically given in Figure 3. Firstly, melamine foam was immersed in HA/Chitosan (CS) composite slurry until the sponge was full of the slurry **(A)**. Then, the melamine foam full of the slurry was transferred into the vacuum oven. During vacuum drying, with the evaporation of the solvent, water, the volume of the filled slurry reduced, so the vacancy uniformly appeared in the foam. Vacuum suction guided the gradual and uniform expansion of vacancy during the evaporation of the solvent **(B)**. At last, the expanding vacancy connected with each other and formed a through-hole structure in the foam. During the solvent evaporation, CS molecular chains shrunk. Because of the strong hydrogen bonding between CS molecular chains and nano-HA, the shrinking of CS molecular chains dragged the nano-HA and made it tightly stuck to the frame of the melamine foam. Thus, uniform through-hole structure was formed in the melamine foam. The schematic diagram was shown in **(B)**. After sintering in air atmosphere, melamine foam and CS were burned out and pure porous HA ceramics were obtained as shown in **(C)**. During sintering, nano-HA was sintered together and porous HA ceramic formed. Simultaneously, the micropores formed on the through-hole structure because of the space occupied by needle-like nano-HA. After the introduction of GO, large GO sheets attached on the surface of the through-hole structure and small GO sheets embedded on the walls of the pores and wrapped into the internal of the micropores because of Van der Waals force **(D)**. Then, thermal reduction was carried out at 1000 °C under nitrogen atmosphere. After heat reduction, the reduced graphene sheets closely integrated with HA because of the shape changes of GO sheets during the reduction and the electrostatic interaction between graphene sheets and HA **(E)** (Zhou et al., 2019).

As a bioceramic material, hydroxyapatite (HA), the main component of the inorganic part of bone in the human skeleton, can be firmly bonded to natural bone and has biological activity to promote bone regeneration and thus has promising applications in bone defect repair. However, its poor tensile strength and crack resistance limit its application in bone tissue engineering. The incorporation of graphene material in the preparation of HA material can improve its mechanical strength without destroying its structure and biocompatibility. It has been reported that the hardness and Young’s modulus were significantly improved in graphene-HA composites (Li et al., 2017). Another study found that the complex of HA combined with rGO can greatly enhance the osteogenic differentiation of cells and new bone formation (Dinescu et al., 2014). If GO is modified by sulfate groups, it can further promote calcium ion aggregation and drive HA mineralization (Fan et al., 2014). HA with a hierarchical pore structure is beneficial for cell adhesion, fluid transfer, and cell ingrowth. The composite scaffolds of hierarchical porous HA combined with rGO can improve adhesion and promote the proliferation and spontaneous osteogenic differentiation of bone marrow mesenchymal stem cells, greatly accelerating bone growth in the scaffold and the repair of critical bone defects (Figure 3 inserted) (Zhou et al., 2019).

**FIGURE 4:**
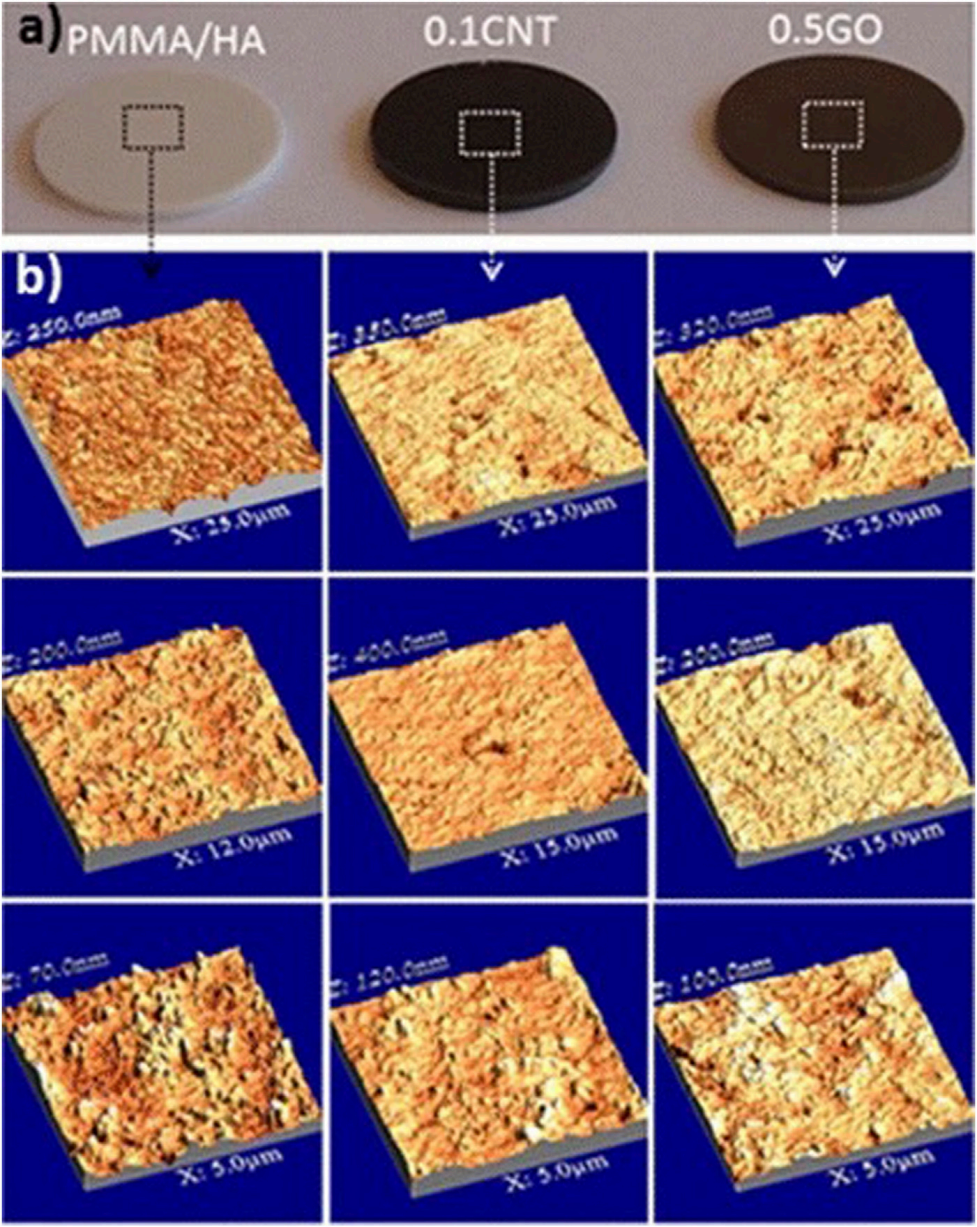
Photograph images of the polished cement disks **(A)** and AFM images of the disks surface recorded at different scales, from 5 to 25 μm **(B)** (Gonçalves et al., 2013).

Poly-methyl methacrylate (PMMA) bone cement is widely used in arthroplasty and vertebroplasty as a molding material with good injectability and high mechanical strength. However, it is not biologically active and cannot form good osseointegration with adjacent bone, which may lead to prosthetic loosening after implantation. In one study, GO at a mass fraction of 0.5% was added to PMMA/HA bone cement. The addition of GO increased the growth of the calcium phosphate layer, enhanced the mechanical properties of the material, and promoted cell proliferation on its surface (Gonçalves et al., 2013) (Figure 4 inserted). These results together increased the binding of the bone cement to the adjacent bone and reduced the risk of potential postoperative prosthetic loosening.

Ultrahigh molecular weight polyethylene (UHMWPE) is widely used as prosthetic liners for artificial hip and knee replacements because of its good biocompatibility, low coefficient of friction, and high impact toughness. However, its low surface hardness produces polyethylene abrasive debris in long-term use, which leads to periprosthetic osteolysis and is an important cause of aseptic loosening of the prosthesis after arthroplasty. By adding a thin layer of 0.1% graphene in the preparation of UHMPE, Lahiri *et al.* increased the fracture toughness and tensile strength of the composite by 54% and 71%, respectively. This finding could help reduce aseptic loosening after arthroplasty (Lahiri et al., 2012).

### 1.3 Progress in the application of graphene and its derivatives in cartilage tissue engineering

Human cartilage tissue is composed of chondrocytes, fibers, and extracellular matrix. Unlike other tissues, cartilage tissue is difficult to recover quickly on its own once damaged because of its lack of vessels and other cells. Currently, cellular therapy using MSCs can induce chondrocyte differentiation and regeneration and is now widely used to regenerate cartilage tissue. In recent years, graphene has been used as a complex scaffold for cartilage stem cell therapy by taking advantage of its ability to stimulate cell growth and differentiation and its excellent mechanical properties.

**FIGURE 5:**
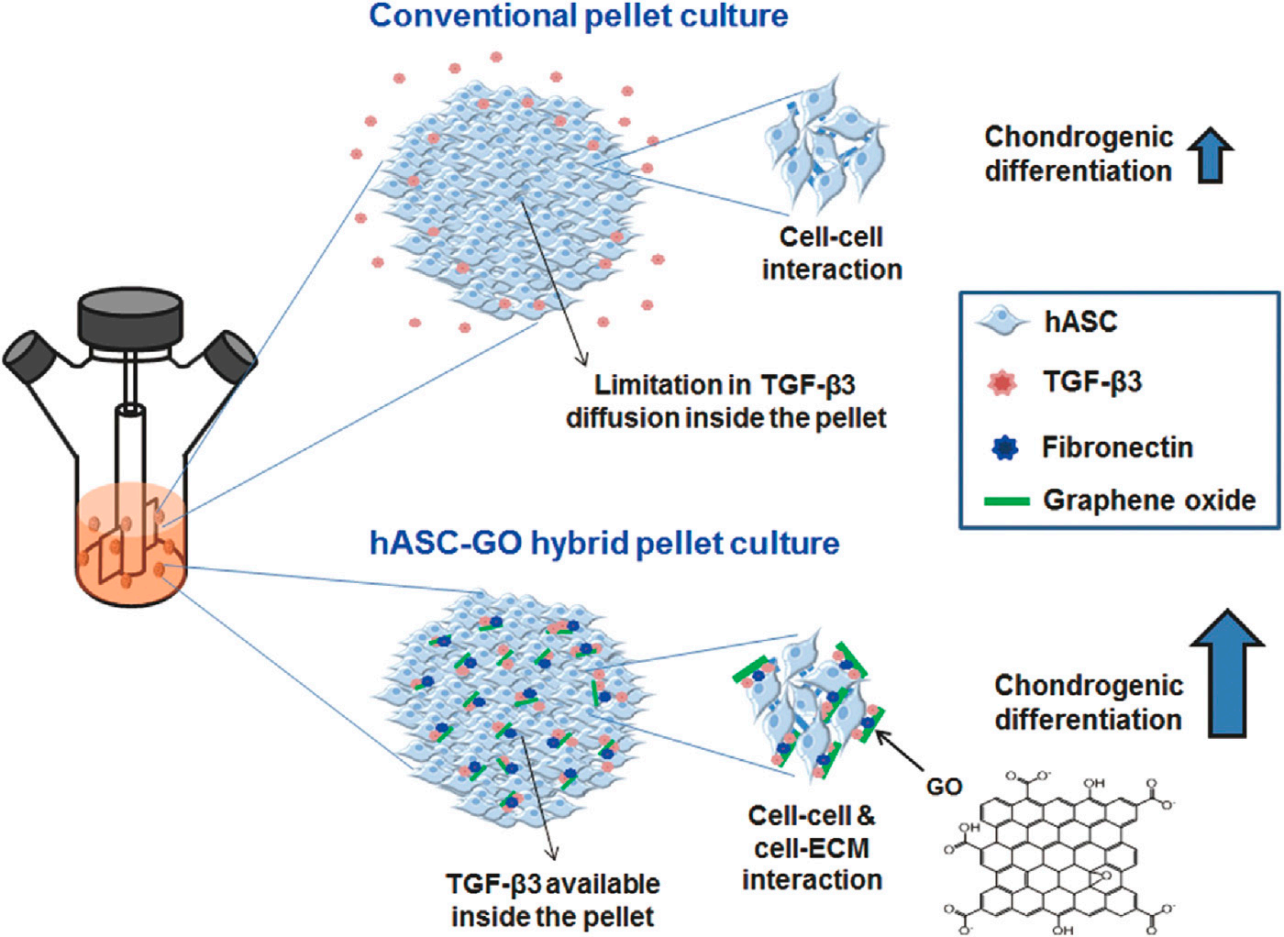
A schematic diagram describing the enhancement in chondrogenic differentiation of hASCs using GO. Conventional pellet culture provides only cell‒cell interactions, and TGF-β3 diffusion inside the pellets is often limited; both of these factors limit the chondrogenic differentiation of stem cells. To improve chondrogenic differentiation, stem cells can be cultured in hybrid pellets of hASCs and GO. GO sheets are adsorbed with cell-adhesion proteins (e.g., FN) and TGF-β3 and dispersed in hASC pellets, providing cell-ECM interactions and TGF-β3 to enhance the chondrogenic differentiation of hASCs (Yoon et al., 2014).

In cartilage tissue engineering, graphene biomaterials play the role of a “growth factor factory”. With the high nanoscale porosity of graphene and its excellent protein-bearing capacity, aggregated proteoglycans, type II collagen, and aminoglucan are assembled with MSCs and graphene sheets to form graphene-cellular biocomposites. Meanwhile, GO can also adsorb fibronectin (FN) and TGF-β proteins through π-π and electrostatic interactions without damaging the protein structure. This composite can improve cell survival as well as chondrocyte differentiation (Figure 5 inserted) (Yoon et al., 2014).

In addition, a cartilage scaffold (CSMA/PECA/GO), synthesized from chondroitin sulfate, ethylene glycol, and GO, has been used to provide a bionic three-dimensional environment. Liao *et al.* found that chondrocytes grown on this composite scaffold had an extremely high survival rate, demonstrating the biocompatibility of the GO composite scaffold. In animal models, when the CSMA/PECA/GO composite scaffold was implanted into models with cartilage tissue defects, better cartilage morphology, more continuous subchondral bone and a thicker neochondral layer were discovered compared to the normal scaffold group (Liao et al., 2015), suggesting that the CSMA/PECA/GO composite scaffold has good prospects in cartilage tissue engineering.

### 1.4 Progress in the application of graphene and its derivatives in nanobiocatalysis

In recent years, much research on nanobiocatalysis has revealed the great ability of nanomaterials to enhance the efficiency and stability of biocatalysts. Nanomaterial-based enzyme mimics (nanozymes), of all nanobiocatalysts, have gained great attention in clinical medicine, bioengineering, pharmaceuticals and many other fields. However, it is still difficult to control nanozymes to target and work more specifically *in vivo* (Liao et al., 2015; Fan et al., 2018). With graphene and its derivatives, researchers have developed nanozymes that can be well regulated and controlled intracellularly.

**FIGURE 6:**
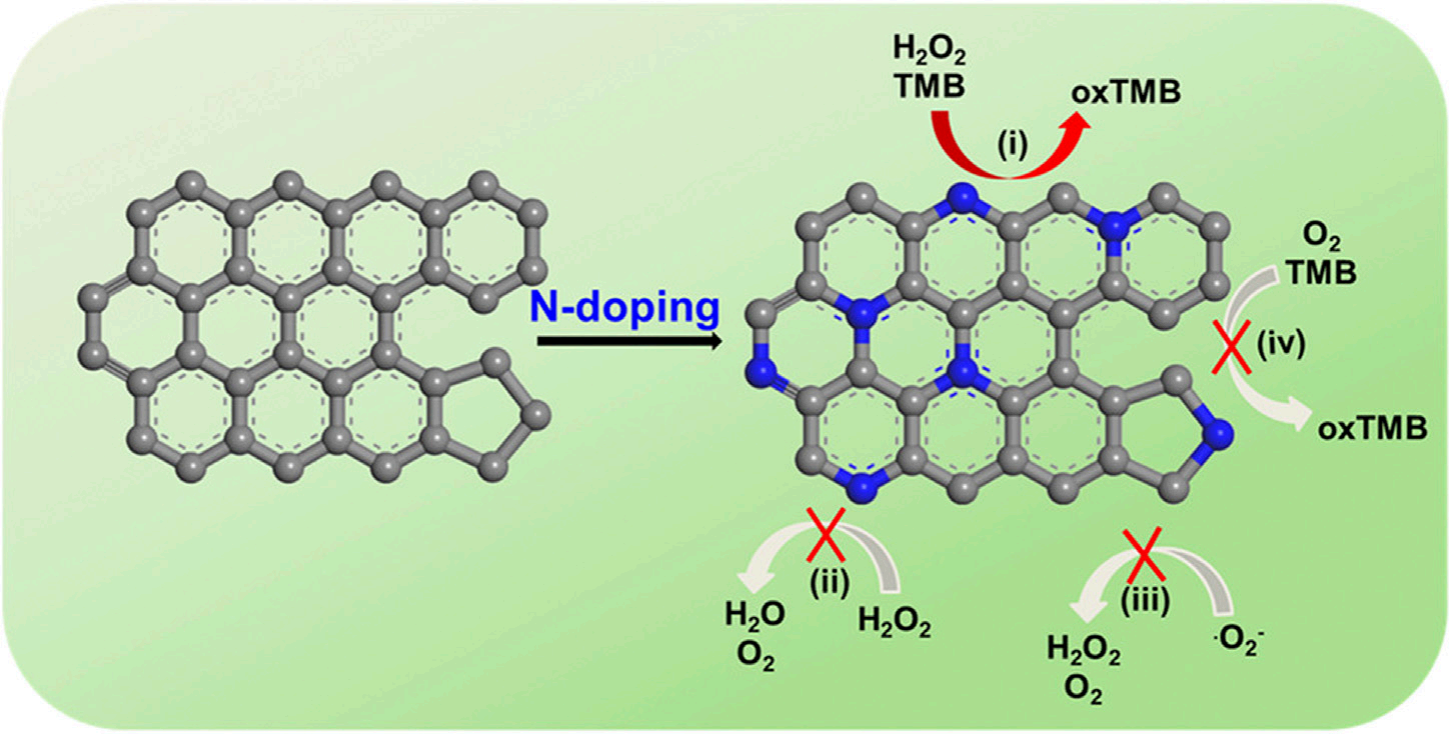
N-rGO exhibits specifically enhanced peroxidase-mimicking activity (i) but negligible catalase-, SOD-, and oxidase-mimicking activities (ii, iii, and iv, respectively). TMB: 3,3′,5,5′-tetramethylbenzidine dihydrochloride hydrate; oxTMB: oxidized TMB (Hu et al., 2018).

Liang *et al.* discovered that nitrogen (N), boron (B), phosphorus (P) and sulfur (S)-doped graphene materials have significant peroxidase-mimicking activity. Furthermore, the dual-doped graphene of P and N shows better mimicking activity because it increases the number of activity sites and has a synergistic effect (Fan et al., 2018). N-doped rGO (N-rGO) also demonstrates enhanced peroxidase-mimicking activities, with no great influence on oxidase-, superoxide dismutase-, or catalase-mimicking activities (Figure 6 inserted) (Hu et al., 2018). These N-doped graphene nanozymes have good biocompatibility and great stability, which is a promising strategy for tumor catalytic therapy (Hu et al., 2018; Liang et al., 2020). When loaded in isabgol nanocomposite scaffolds, rGO can heal wounds rapidly with magnified angiogenesis, collagen synthesis and deposition, especially in diabetic wounds (Thangavel et al., 2018). Qiu *et al.* also reported that graphene-based materials, especially those encapsulated with nanoparticles such as TiO_2_, have a great ability to protect against oxidation (Qiu et al., 2014). In immunological regulation, graphene quantum dots (GQDs) have received much attention for their great anti-inflammatory properties for treating intestinal bowel diseases (IBDs) (Lee et al., 2020).

**FIGURE 7:**
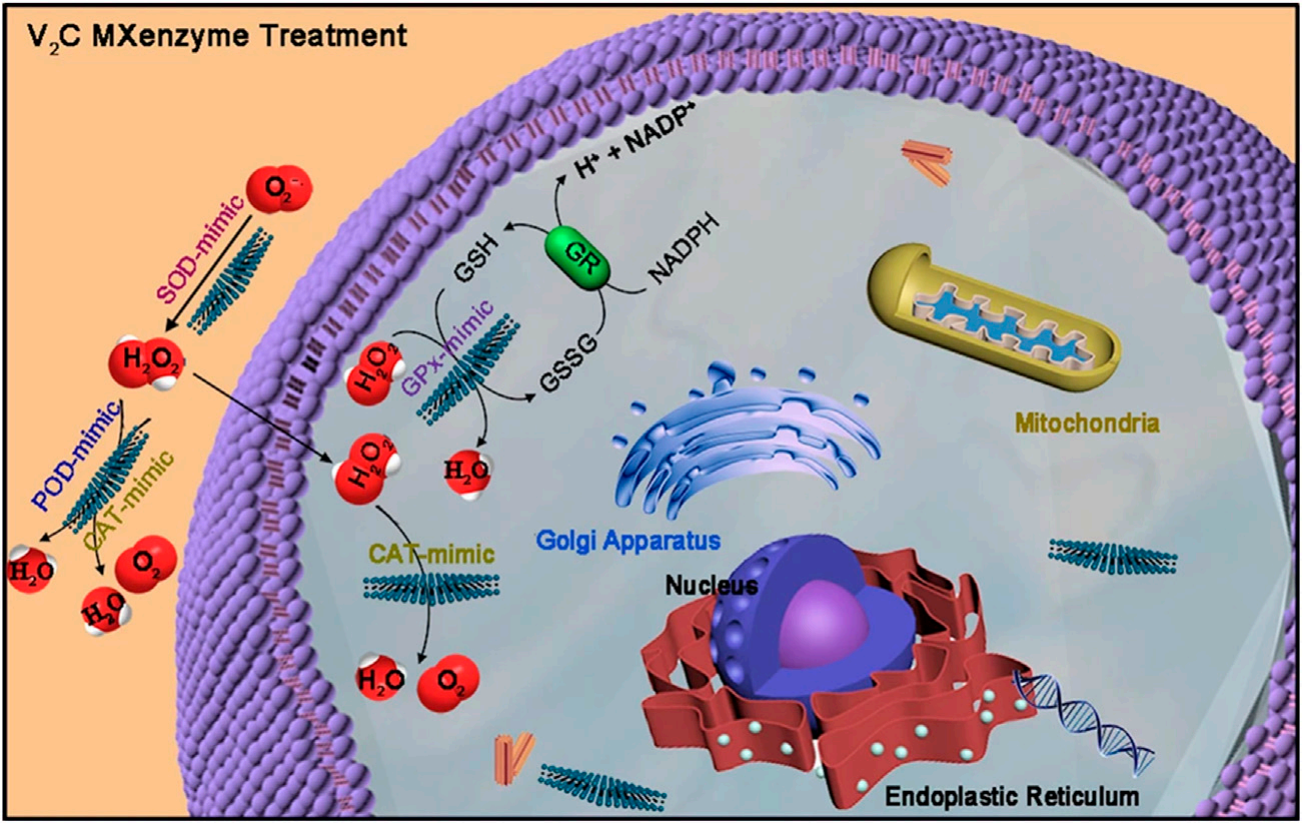
V2C MXenzyme effectively catalyzes O_2_−˙ into H_2_O_2_ and O_2_, decomposes H_2_O_2_ into O_2_ and H_2_O, and gets rid of ˙OH (Feng W. et al., 2021).

Meanwhile, researchers discovered that other carbon materials might also play an important role in nanobiocatalysis. Metal carbides and/or nitrides (MXenes), a series of two-dimensional (2D) nanomaterials, have great physicochemical properties, including high metallic conductivity, excellent hydrophilicity and a large surface area (Liu et al., 2022). Feng *et al.* reported that a two-dimensional (2D) vanadium carbide (V2C) MXene nanoenzyme (MXenzyme) showed oxidase, peroxidase, superoxide dismutase, and catalase activities similar to those of natural enzymes (Figure 7 inserted) (Feng W. et al., 2021). Likewise, reactive oxygen species (ROS)-based nanomaterials, which are deeply related to cell signaling and tissue homeostasis, can scavenge free radicals, protecting cells against oxidative stress and leading to a possible treatment strategy (Zhou et al., 2020; Zhao et al., 2022). Similarly, Ali *et al.* found that a tris-malonic acid derivative of fullerene (C_60_) showed approximate properties to superoxide dismutase (Ali et al., 2004), and the hydrogel of fullerene can be utilized to treat cardiac damage (Hao et al., 2017).

**FIGURE 8:**
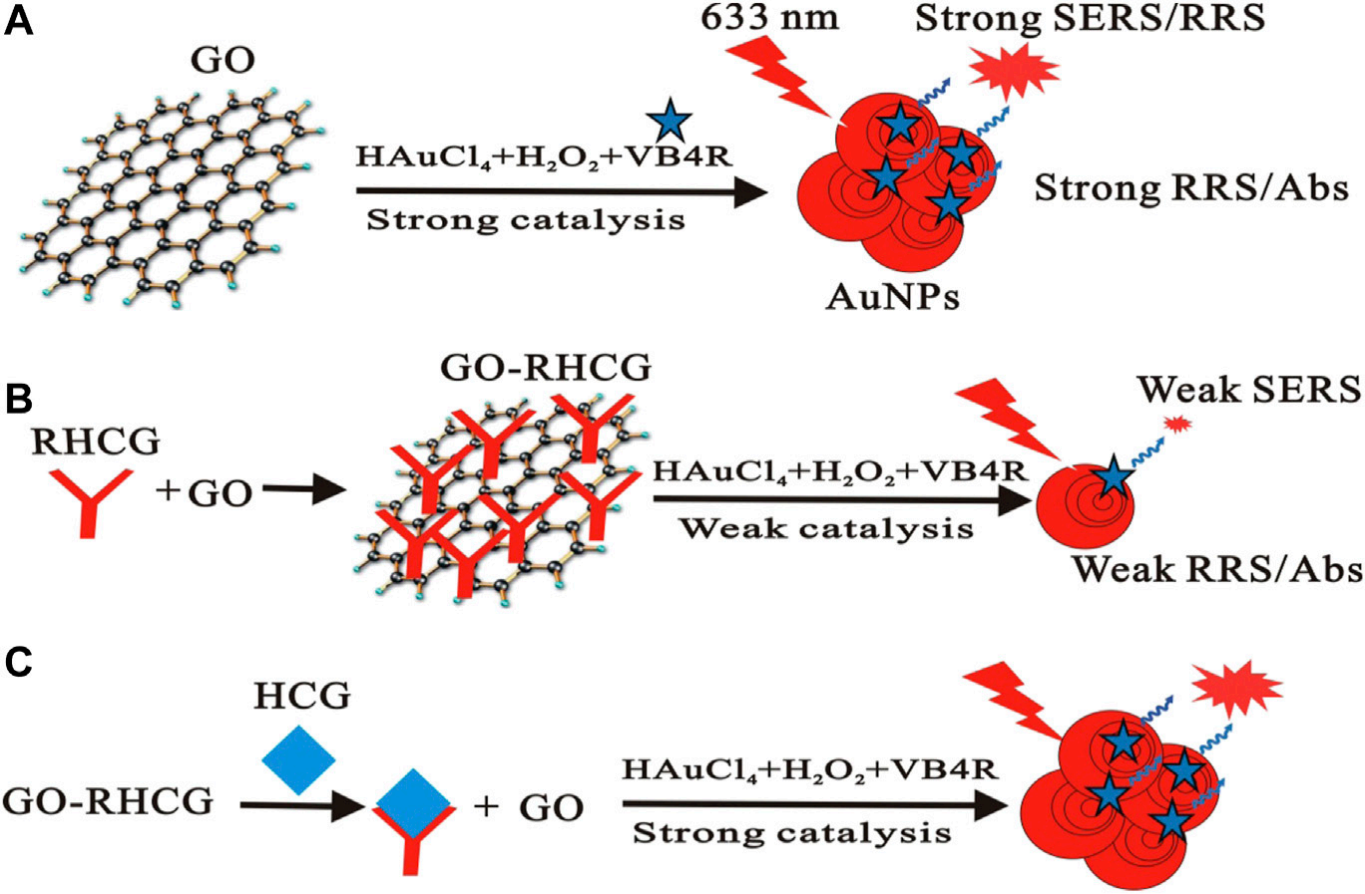
Scheme of the immunecontrolling GO catalytic activity–SERS detection of HCG. GO catalyzed the formed AuNPs with strong SERS **(A)**. RHCG inhabited the nanocatalytic reaction with weak SERS **(B)**. HCG recovered the nanocatalysis to form AuNPs with strong SERS **(C)** (Liang et al., 2017).

Apart from being used as a nanozyme, GO also has surface-enhanced Raman scattering (SERS) activity (Figure 8 inserted) (Liang et al., 2017). More GO added forms more gold nanoparticles (AuNPs) in the gold nanoreaction between HAuCl_4_ and H_2_O_2_, resulting in linear enhancement of SERS, resonance Rayleigh scattering (RRS), and surface plasmon resonance (SPR) absorptions. This new strategy of immunocontrolling GO catalysis has been well adapted in the detection of HCG. The above discoveries of graphene and its derivatives being utilized in both clinical treatment and laboratory tests suggest more application in nanobiocatalysis.

## 2 Conclusion

This review presented a wholesome picture of graphene and its derivatives, from their basic information to their special biological properties, including antibacterial ability, bone tissue regeneration promotability and drug delivery capacity. The application of graphene and its derivatives was then emphatically elaborated in bone and cartilage tissue engineering and nanobiocatalysis.

With a variety of unique biological functions, such as drug-carrying properties, antibacterial properties and osteoblast differentiation, graphene has not only become a new direction in the field of bone and cartilage tissue engineering but is also expected to bring new breakthroughs in clinical treatment. Although graphene has received widespread attention, it is still in its infancy, especially for the interaction between graphene and various cells, tissues and organs, and there is still a lack of systematic and safe research. In the future, much basic and clinical research is still needed to break through the gap of graphene safety and degradability, making it a new application material in the field of bone and cartilage tissue engineering.

## References

[B1] Ali S. S. Hardt J. I. Quick K. L. Sook Kim-Han J. Erlanger B. F. Huang T. t. (2004). A biologically effective fullerene (C60) derivative with superoxide dismutase mimetic properties. Free Radic. Biol. Med. 37, 1191–1202. 10.1016/j.freeradbiomed.2004.07.002 15451059

[B2] Boccaccini A. R. Gerhardt L. C. (2010). Carbon nanotube composite scaffolds and coatings for tissue engineering applications. Key Eng. Mater. 441, 31–52. 10.4028/www.scientific.net/kem.441.31

[B3] Dinescu S. Ionita M. Pandele A. M. Galateanu B. Iovu H. Ardelean A. (2014). *In vitro* cytocompatibility evaluation of chitosan/graphene oxide 3D scaffold composites designed for bone tissue engineering. Bio-Med. Mater. Eng. 24, 2249–2256. 10.3233/bme-141037 25226924

[B4] Fan Z. Wang J. Wang Z. Ran H. Li Y. Niu L. (2014). One-pot synthesis of graphene/hydroxyapatite nanorod composite for tissue engineering. Carbon 66, 407–416. 10.1016/j.carbon.2013.09.016

[B5] Fan K. Xi J. Fan L. Wang P. Zhu C. Tang Y. (2018). *In vivo* guiding nitrogen-doped carbon nanozyme for tumor catalytic therapy. Nat. Commun. 9, 1440. 10.1038/s41467-018-03903-8 29650959 PMC5897348

[B6] Feng B. Zhu W. Bian Y. Y. Chang X. Cheng K. Y. Weng X. S. (2021). China artificial joint annual data report. Chin. Med. J. 134, 752–753. 10.1097/cm9.0000000000001196 PMC7990009 33725711

[B7] Feng W. Han X. Hu H. Chang M. Ding L. Xiang H. (2021). 2D vanadium carbide MXenzyme to alleviate ROS-mediated inflammatory and neurodegenerative diseases. Nat. Commun. 12, 2203. 10.1038/s41467-021-22278-x 33850133 PMC8044242

[B8] Gao C. Liu T. Shuai C. Peng S. (2014). Enhancement mechanisms of graphene in nano-58S bioactive glass scaffold: Mechanical and biological performance. Sci. Rep. 4, 4712–4810. 10.1038/srep04712 24736662 PMC3988481

[B9] Geim A. K. Novoselov K. S. (2007). The rise of graphene. Nat. Mater. 6, 183–191. 10.1038/nmat1849 17330084

[B10] Gonçalves G. Portolés M. T. Ramírez-Santillán C. Vallet-Regí M. Serro A. P. Grácio J. (2013). Evaluation of the *in vitro* biocompatibility of PMMA/high-load HA/carbon nanostructures bone cement formulations. J. Mater. Sci. Mater. Med. 24, 2787–2796. 10.1007/s10856-013-5030-2 23963685

[B11] Gurunathan S. Han J. W. Dayem A. A. Eppakayala V. Park M. R. Kwon D. N. (2013). Antibacterial activity of dithiothreitol reduced graphene oxide. J. Indust. Eng. Chem. 19, 1280–1288. 10.1016/j.jiec.2012.12.029

[B12] Hao T. Li J. Yao F. Dong D. Wang Y. Yang B. (2017). Injectable fullerenol/alginate hydrogel for suppression of oxidative stress damage in Brown adipose-derived stem cells and cardiac repair. ACS Nano 11, 5474–5488. 10.1021/acsnano.7b00221 28590722

[B13] Hu W. Peng C. Luo W. Lv M. Li X. Li D. (2010). Graphene-based antibacterial paper. ACS Nano 4, 4317–4323. 10.1021/nn101097v 20593851

[B14] Hu Y. Gao X. J. Zhu Y. Muhammad F. Tan S. Cao W. (2018). Nitrogen-doped carbon nanomaterials as highly active and specific peroxidase mimics. Chem. Mater. 30, 6431–6439. 10.1021/acs.chemmater.8b02726

[B15] Jang J.-Y. Lee S. W. Park S. H. Shin J. W. Mun C. Kim S. H. (2011). Combined effects of surface morphology and mechanical straining magnitudes on the differentiation of mesenchymal stem cells without using biochemical reagents. J. Biomed. Biotechnol. 2011, 1–9. 10.1155/2011/860652 PMC3043320 21403908

[B16] Kalbacova M. Broz A. Kong J. Kalbac M. (2010). Graphene substrates promote adherence of human osteoblasts and mesenchymal stromal cells. Carbon 48, 4323–4329. 10.1016/j.carbon.2010.07.045

[B17] Kang M. S. Jang H. J. Lee S. H. Shin Y. C. Hong S. W. Lee J. H. (2022). Functional graphene nanomaterials-based hybrid scaffolds for osteogenesis and chondrogenesis. Adv. Exp. Med. Biol. 1351, 65–87. 10.1007/978-981-16-4923-3_4 35175612

[B18] Kim J. Choi K. S. Kim Y. Lim K. T. Seonwoo H. Park Y. (2013). Bioactive effects of graphene oxide cell culture substratum on structure and function of human adipose‐derived stem cells. J. Biomed. Mater. Res. Part A Off. J. Soc. Biomaterials 101, 3520–3530. 10.1002/jbm.a.34659 23613168

[B19] Krishnamoorthy K. Veerapandian M. Zhang L.-H. Yun K. Kim S. J. (2012). Antibacterial efficiency of graphene nanosheets against pathogenic bacteria via lipid peroxidation. J. Phys. Chem. C 116, 17280–17287. 10.1021/jp3047054

[B20] Lahiri D. Dua R. Zhang C. de Socarraz-Novoa I. Bhat A. Ramaswamy S. (2012). Graphene nanoplatelet-induced strengthening of ultrahigh molecular weight polyethylene and biocompatibility *in vitro* . ACS Appl. Mater. interfaces 4, 2234–2241. 10.1021/am300244s 22439663

[B21] Lee B.-C. Lee J. Y. Kim J. Yoo J. M. Kang I. Kim J. J. (2020). Graphene quantum dots as anti-inflammatory therapy for colitis. Sci. Adv. 6, eaaz2630. 10.1126/sciadv.aaz2630 32494673 PMC7190325

[B22] Li J. Liu X. Crook J. M. Wallace G. G. (2017). Development of a porous 3D graphene-PDMS scaffold for improved osseointegration. Colloids Surfaces B Biointerfaces 159, 386–393. 10.1016/j.colsurfb.2017.07.087 28818783

[B23] Liang A. Wang X. Luo Y. Wen G. Jiang Z. (2017). Immunocontrolling graphene oxide catalytic nanogold reaction and its application to SERS quantitative analysis. ACS omega 2, 7349–7358. 10.1021/acsomega.7b01335 30023549 PMC6044934

[B24] Liang Q. Xi J. Gao X. J. Zhang R. Yang Y. Gao X. (2020). A metal-free nanozyme-activated prodrug strategy for targeted tumor catalytic therapy. Nano Today 35, 100935. 10.1016/j.nantod.2020.100935

[B25] Liao J. Qu Y. Chu B. Zhang X. Qian Z. (2015). Biodegradable CSMA/PECA/graphene porous hybrid scaffold for cartilage tissue engineering. Sci. Rep. 5, 9879. 10.1038/srep09879 25961959 PMC4426702

[B26] Liu Z. Robinson J. T. Sun X. Dai H. (2008). PEGylated nanographene oxide for delivery of water-insoluble cancer drugs. J. Am. Chem. Soc. 130, 10876–10877. 10.1021/ja803688x 18661992 PMC2597374

[B27] Liu S. Zeng T. H. Hofmann M. Burcombe E. Wei J. Jiang R. (2011). Antibacterial activity of graphite, graphite oxide, graphene oxide, and reduced graphene oxide: Membrane and oxidative stress. ACS Nano 5, 6971–6980. 10.1021/nn202451x 21851105

[B28] Liu J. Lu W. Lu X. Zhang L. Dong H. Li Y. (2022). Versatile Ti 3 C 2 T x MXene for free-radical scavenging. Nano Res. 15, 2558–2566. 10.1007/s12274-021-3751-y 34518776 PMC8427154

[B29] McBeath R. Pirone D. M. Nelson C. M. Bhadriraju K. Chen C. S. (2004). Cell shape, cytoskeletal tension, and RhoA regulate stem cell lineage commitment. Dev. Cell 6, 483–495. 10.1016/s1534-5807(04)00075-9 15068789

[B30] Nayak T. R. Andersen H. Makam V. S. Khaw C. Bae S. Xu X. (2011). Graphene for controlled and accelerated osteogenic differentiation of human mesenchymal stem cells. ACS Nano 5, 4670–4678. 10.1021/nn200500h 21528849

[B31] Nejabat M. Charbgoo F. Ramezani M. (2017). Graphene as multifunctional delivery platform in cancer therapy. J. Biomed. Mater. Res. Part A 105, 2355–2367. 10.1002/jbm.a.36080 28371194

[B32] Novoselov K. S. Geim A. K. Morozov S. V. Jiang D. Zhang Y. Dubonos S. V. (2004). Electric field effect in atomically thin carbon films. Science 306, 666–669. 10.1126/science.1102896 15499015

[B33] Qiu Y. Wang Z. Owens A. C. E. Kulaots I. Chen Y. Kane A. B. (2014). Antioxidant chemistry of graphene-based materials and its role in oxidation protection technology. Nanoscale 6, 11744–11755. 10.1039/c4nr03275f 25157875 PMC4312421

[B34] Ryoo S.-R. Kim Y.-K. Kim M.-H. Min D.-H. (2010). Behaviors of NIH-3T3 fibroblasts on graphene/carbon nanotubes: Proliferation, focal adhesion, and gene transfection studies. ACS Nano 4, 6587–6598. 10.1021/nn1018279 20979372

[B35] Shin S. R. Li Y. C. Jang H. L. Khoshakhlagh P. Akbari M. Nasajpour A. (2016). Graphene-based materials for tissue engineering. Adv. Drug Deliv. Rev. 105, 255–274. 10.1016/j.addr.2016.03.007 27037064 PMC5039063

[B36] Siddiqi A. Levine B. R. Springer B. D. (2022). Highlights of the 2021 American joint replacement registry annual report. Arthroplasty Today 13, 205–207. 10.1016/j.artd.2022.01.020 35128013 PMC8810304

[B37] Thangavel P. Kannan R. Ramachandran B. Moorthy G. Suguna L. Muthuvijayan V. (2018). Development of reduced graphene oxide (rGO)-isabgol nanocomposite dressings for enhanced vascularization and accelerated wound healing in normal and diabetic rats. J. Colloid interface Sci. 517, 251–264. 10.1016/j.jcis.2018.01.110 29428812

[B38] Tu Y. Lv M. Xiu P. Huynh T. Zhang M. Castelli M. (2013). Destructive extraction of phospholipids from *Escherichia coli* membranes by graphene nanosheets. Nat. Nanotechnol. 8, 594–601. 10.1038/nnano.2013.125 23832191

[B39] Yang X. Tu Y. Li L. Shang S. Tao X.-m. (2010). Well-dispersed chitosan/graphene oxide nanocomposites. ACS Appl. Mater. interfaces 2, 1707–1713. 10.1021/am100222m 20527778

[B40] Yin S. Goldovsky Y. Herzberg M. Liu L. Sun H. Zhang Y. (2013). Functional free‐standing graphene honeycomb films. Adv. Funct. Mater. 23, 2972–2978. 10.1002/adfm.201203491

[B41] Yoon H. H. Bhang S. H. Kim T. Yu T. Hyeon T. Kim B. S. (2014). Dual roles of graphene oxide in chondrogenic differentiation of adult stem cells: Cell‐adhesion substrate and growth factor‐delivery carrier. Adv. Funct. Mater. 24, 6455–6464. 10.1002/adfm.201400793

[B42] Zhang L. Xia J. Zhao Q. Liu L. Zhang Z. (2010). Functional graphene oxide as a nanocarrier for controlled loading and targeted delivery of mixed anticancer drugs. small 6, 537–544. 10.1002/smll.200901680 20033930

[B43] Zhao T. Wu W. Sui L. Huang Q. Nan Y. Liu J. (2022). Reactive oxygen species-based nanomaterials for the treatment of myocardial ischemia reperfusion injuries. Bioact. Mater. 7, 47–72. 10.1016/j.bioactmat.2021.06.006 34466716 PMC8377441

[B44] Zhou K. Yu P. Shi X. Ling T. Zeng W. Chen A. (2019). Hierarchically porous hydroxyapatite hybrid scaffold incorporated with reduced graphene oxide for rapid bone ingrowth and repair. ACS Nano 13, 9595–9606. 10.1021/acsnano.9b04723 31381856

[B45] Zhou Z. Ni K. Deng H. Chen X. (2020). Dancing with reactive oxygen species generation and elimination in nanotheranostics for disease treatment. Adv. Drug Deliv. Rev. 158, 73–90. 10.1016/j.addr.2020.06.006 32526453

