# Corrigendum: Progress of research on Graphene and its derivatives in bone and cartilage repair

**DOI:** 10.3389/fbioe.2023.1268642

**Published:** 2023-10-10

**Authors:** Shilong Yu, Mingke You, Kai Zhou, Jian Li

**Affiliations:** ^1^ West China School of Medicine, West China Hospital, Sichuan University, Chengdu, China; ^2^ Sports Medicine Center, West China Hospital, Sichuan University, Chengdu, China; ^3^ Department of Orthopedics, Orthopedic Research Institute, West China Hospital, Sichuan University, Chengdu, China

**Keywords:** graphene-based materials, bone, cartilage, tissue engineering, nanobiocatalysis

In the published article, there was a mistake in the **Corresponding Author** annotation. The corresponding author annotation displayed both “Jian Li and Kai Zhou” as corresponding authors. However, “Kai Zhou” should be the only corresponding author for the article.

The authors apologize for this error and state that this does not change the scientific conclusions of the article in any way. The original article has been updated.

